# Can we use the questionnaire SNOT-22 as a predictor for the indication of surgical treatment in chronic rhinosinusitis?^☆^^☆☆^

**DOI:** 10.1016/j.bjorl.2016.05.010

**Published:** 2016-06-24

**Authors:** Pablo Pinillos Marambaia, Manuela Garcia Lima, Marina Barbosa Guimarães, Amaury de Machado Gomes, Melina Pinillos Marambaia, Otávio Marambaia dos Santos, Leonardo Marques Gomes

**Affiliations:** a Escola Bahiana de Medicina e Saúde Pública (Bahiana), Programa de Pós-graduação, Salvador, BA, Brazil; b Universidade Federal da Bahia (UFBA), Salvador, BA, Brazil; c Instituto de Otorrinolaringologia Otorrinos Associados (INOOA), Salvador, BA, Brazil; d Santa Casa de São Paulo, Otorrinolaringologia, São Paulo, SP, Brazil; e Universidade do Porto, Bioética, Porto, Portugal; f Universidade Federal de São Paulo (UNIFESP), Programa de Pós-graduação em Otorrinolaringologia, São Paulo, SP, Brazil

**Keywords:** Nasal surgical procedures, Quality of life, Sinusitis, Procedimentos cirúrgicos nasais, Qualidade de vida, Sinusite

## Abstract

**Introduction:**

Chronic rhinosinusitis is a prevalent disease that has a negative impact on the lives of sufferers. SNOT-22 is considered the most appropriate questionnaire for assessing the quality of life of these patients and a very effective method of evaluating therapeutic interventions; however it is not used as a tool for decision-making.

**Objective:**

To test the hypothesis that the SNOT-22 score can predict the outcome of surgical treatment.

**Methods:**

A retrospective, longitudinal and analytical study. We evaluated the medical records of patients with chronic rhinosinusitis that completed the SNOT-22 at the time of diagnosis. All the patients were consecutively receiving care at an otolaryngology service in Salvador, Bahia from August 2011 to June 2012. The outcomes of the surgical treatment of these patients were obtained from their medical records. The initial score was compared to a group of patients who were not referred for surgery. All the patients completed and signed a consent form.

**Results:**

Of the 88 patients with chronic rhinosinusitis, 26 had evolved to surgery over the last 3 years. The groups were homogeneous regarding gender and respiratory and medication allergies. The patients of the surgical group were 44.8 + 13.8 years old and the patients of the clinical group were 38.2 + 12.5 years old (*p* = 0.517). The average SNOT-22 score of the case group was 49 + 19 and the average score of the control group was 49 + 27 (*p* = 0.927).

**Conclusion:**

The SNOT-22 was unable to predict the outcome of surgical patients with chronic rhinosinusitis.

## Resumo

**Introdução:**

A rinossinusite crônica é uma doença prevalente que tem um impacto negativo sobre a vida dos portadores. O SNOT-22 é considerado o questionário mais adequado para avaliar a qualidade de vida desses pacientes e um método muito eficaz de avaliar intervenções terapêuticas; no entanto, ele não é usado como uma ferramenta para a tomada de decisões.

**Objetivo:**

Testa a hipótese de que o escore do SNOT-22 pode prever o desfecho do tratamento cirúrgico.

**Método:**

Estudo retrospectivo, longitudinal e analítico. Foram avaliados os prontuários de pacientes com rinossinusite crônica que preencheram o SNOT-22 no momento do diagnóstico. Todos os pacientes foram consecutivamente atendidos em um serviço de otorrinolaringologia em Salvador, Bahia, de agosto de 2011 a junho de 2012. Os desfechos do tratamento cirúrgico desses pacientes foram obtidos a partir de seus prontuários médicos. A pontuação inicial foi comparada com um grupo de pacientes que não foi encaminhado para cirurgia. Todos os pacientes preencheram e assinaram um termo de consentimento informado.

**Resultados:**

Dos 88 pacientes com rinossinusite crônica, 26 evoluíram para cirurgia nos últimos três anos. Os grupos foram homogêneos quanto a sexo, alergias respiratórias e medicamentos. Os pacientes do grupo cirúrgico tinham 44,8 + 13,8 anos e os do grupo clínico tinham 38,2 + 12,5 (p = 0,517). O escore médio do SNOT-22 do grupo do caso foi de 49 + 19 e o escore médio do grupo controle foi de 49 + 27 (p = 0,927)

**Conclusão:**

O SNOT-22 foi incapaz de prever o desfecho dos pacientes cirúrgicos com rinossinusite crônica.

## Introduction

Data on the quality of life of patients with chronic rhinosinusitis (CRS) prove that this disease has a major impact on the activities of daily living of these patients.

It has already been proved that CRS negatively affects the QOL of sufferers in comparison to people without the disease and people with other chronic diseases like congestive heart failure and chronic obstructive pulmonary disease.^1^

The main focus of these studies was the use of questionnaires to evaluate the impact of therapeutic interventions. The same questionnaire is generally applied before and after intervention to a group of patients. The impact of surgery on the betterment of patients with CRS has been exhaustively studied and there seems to be a consensus, especially in the short-term assessment.^2^ Studies show that the improvement rates of surgery range from 76% to 97.5%.^3^, ^4^

The SinoNasal Outcome Test 22 (SNOT-22) is an easily applied questionnaire that has been validated for use in Portuguese.^5^ This instrument has 22 questions about possible symptoms linked to chronic rhinosinusitis. Each question receives a score from 0 to 5, where zero is the absence of this condition and five is the worst possible case of this condition. Similarly, higher total scores represent a worse quality of life. According to the 2012 European Position Paper on Rhinosinusitis and Nasal Polyps (EPOS), SNOT-22 is a good tool for assessing QOL in patients with CRS. Moreover, it can be used repeatedly and produces graphics (SNOTgrams) with SNOT-22 scores for more than a given moment in time, which clearly display the result of medicinal and surgical interventions and exacerbations over time.^6^

Since the 1990s, the benefit of functional endoscopic surgery of the paranasal sinuses has been demonstrated by assessing specific symptoms such as nasal obstruction, for example. Later, QOL became an additional parameter in this assessment and several studies have used this tool to evaluate patients. This practice has led to the hope that we can extrapolate the use of the questionnaires mostly to select patients for different types of treatment and determine how to interpret the information of populations outside Brazil and apply it to our scenario.

The criteria of surgical intervention, for example, are poorly described in literature and consequently lead to a broad geographical variation of this indication and a loss of quality in medical care.^7^ Measures that can standardise or facilitate this decision would help improve the follow-up of these sufferers.

The present study aims to compare the average score of the SNOT-22 in the initial assessment of patients with chronic rhinosinusitis and test the hypothesis that the SNOT-22 score can predict the outcome of surgical therapy.

## Methods

This is a descriptive and analytical retrospective longitudinal study with a convenience sample derived from a previous study of the same author.

We accessed records of the patients who participated in the previous study. These patients had received care for the first time between 2011 and 2012 and continued supervised care at an otolaryngology service in Salvador, Bahia, until August 2015.

The inclusion criteria were literate patients with chronic rhinosinusitis over 18 years of age.

The diagnosis of chronic rhinosinusitis was determined using the criteria of the EPOS-2012,^6^ whereby chronic rhinosinusitis is defined by the presence of two or more symptoms of nasal obstruction/congestion/blockage, anterior or posterior rhinorrhea, anosmia or hyposmia/anosmia and facial pain/pressure for more than 12 weeks that must be the result of nasal obstruction/congestion/blockage or anterior or posterior purulent rhinorrhea.

The criteria for exclusion were illiterate patients, smokers, patients with immune deficiency, cystic fibrosis or primary ciliary dyskinesia, patients with benign or malignant nasal tumours, patients with granulomatous diseases and vasculitis, patients who had previously undergone surgery and subjects who refused to participate in the study.

All the patients were evaluated during the first consultation and after the confirmation of CRS. The patients subsequently completed a registration form with demographic data, the SNOT-22 questionnaire validated for Portuguese^8^ and an informed consent statement.

The SNOT-22 questionnaire was applied during the first consultation when the patients were evaluated by the same professional, in 2011 and 2012.

After 3 years, the medical records were reviewed to verify the referral for clinical or surgical treatment over time. This referral was made by the same ENT professional who was blinded to the SNOT-22 score.

The subjects were divided into two groups: The group that evolved to the referral for surgery during the studied period and the group that continued with clinical treatment.

Surgery was referred after maximum clinical treatment had failed for at least 3 weeks. Maximum clinical treatment is defined as the use of topical or systemic corticosteroids, antibiotic therapy and saline nasal irrigation.

The failure of clinical treatment was defined as the lack of improvement in symptoms referred by the actual patient. In the absence of a response, an assessment computed tomography was requested, as well as possible scheduling of a future surgery.

Surgery was also indicated when tomographic analysis led to the diagnosis of a condition that required surgical treatment, namely significant anatomical changes such as obstructive septum deviation, large or obstructive middle turbinate pneumatisation or extensive sinonasal polyposis, and rhinosinusitis of dental or fungal origin.

Furthermore, surgery was indicated according to the mentioned criteria and the conduct of a single professional, although not all the patients were necessarily operated since elements such as motivation, personal preference and expectations regarding the procedure influenced the decision.

This study was approved by the Ethics Committee of the institution, under protocol n° 181/2011.

### Data analysis

The sample size was calculated using WinPepi version 11.62, with a standard deviation of the SNOT-22 score of a previous Brazilian study involving surgical patients (DP = 25), Kosugi et al.,^8^ to detect a difference of 20 points. In this case, 50 patients would be needed, divided into two groups of 25 subjects. Consequently, the sample of the present study exceeds the required number of participants.

The results were tabulated and analysed using SPSS-17 software.

The categorical demographic data like gender and presence of comorbidities and allergies were arranged using the valid percentile. The chi-square test was used to compare categorical variables between the groups.

The score of the SNOT-22 questionnaire was described using the average and standard deviation since the sample distribution was normal.

The averages between the groups were compared using the unpaired *t*-test.

The unpaired *t*-test was also used to compare the average score of each item of the SNOT-22 individually.

The alpha error was considered acceptable when the value of *p* < 0.05.

## Results

A total of 88 patients were analysed, of which 26 were patients referred for surgery and 62 evolved to drug therapy.

Table 1 shows the demographic characteristics of the sample.

**Table 1:** Sociodemographic characteristics of patients with chronic rhinosinusitis referred for surgery (surgery group) and of patients with chronic rhinosinusitis referred for clinical treatment (clinical group).

Variables	Surgery group (*n* = 26)	Clinical group (*n* = 62)	Significance (*p*)
*Gender (%)*			
Male	12 (41)	24 (40.8)	0.517
Female	14 (59)	38	0.517

*Age (years)*	44.8 + 13.8	38.2 + 12.5	0.438

*Comorbidities*			
SAH	03	02	0.81
DM	0	02	0.307
Asthma	04	02	0.167

*Allergy to medication (%)*			
Yes	07 (22.8)	12 (13.3)	0.594
No	19 (77.2)	50 (86.7)	

*Respiratory allergy (%)*			
Yes	02 (7.7)	05 (8.1)	0.810
No	24 (92.3)	57 (91.9)	

Surgery group, patients referred for surgery; clinical group, patients referred for clinical treatment.

Significance level *p* < 0.05.

**Figure 1:**
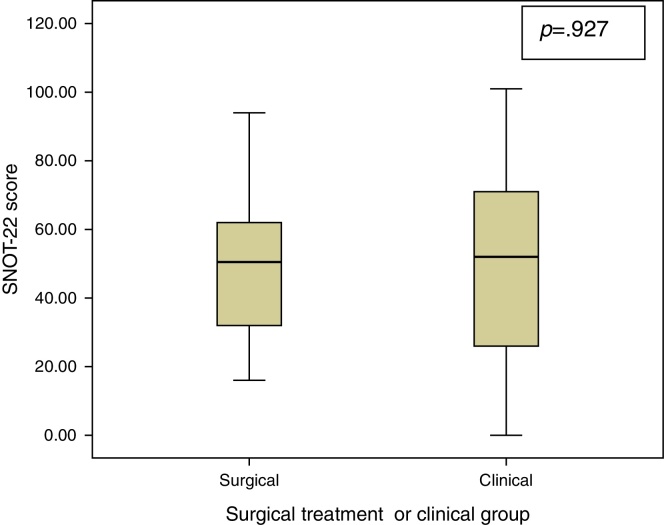
Shows the comparison of the SNOT-22 score averages of the groups.

With regard to the SNOT-22 score in the first consultation, it was found that the group that evolved to surgical treatment scored 49.4 ± 19.8 and clinical group averaged 49.9 ± 27 (Table 2 and Fig. 1).

**Table 2:** Quality of life score with SNOT-22 of the groups.

Variable	Surgery group	Clinical group	Significance (*p*)
SNOT-22	49 (±19)	49 (±27)	0.927

SNOT-22, Sino Nasal Outcome Test.

Significance level *p* < 0.05. Unpaired *t*-test. Average (standard deviation).

The comparison of each item (symptom) of the SNOT-22 questionnaire did not show any difference between the groups.

## Discussion

The QOL assessment of patients with CRS requires specific questionnaires to measure the results, such as those obtained after interventions with medication and surgery. A vast amount of studies use these instruments to assess surgical treatment^3^, ^4^ and some authors believe that questionnaires can provide additional information for diagnoses and decision making.^9^

Soler et al.^9^ also reported that a low score of the questionnaire was the only factor that was related to the decision to undergo surgery and concluded that questionnaires to assess quality of life should be incorporated into clinical practice.

Smith et al.^10^ conducted a prospective study that showed that patients with worse scores benefit more from surgery. Moreover, patients with clinical monitoring and worse quality of life scores could switch to the surgical group, which led to a significant improvement of the scores.

Birch et al.^11^ suggest that patients who are waiting for surgery should have worse endoscopic scores, more CRS symptoms and worse QOL scores.

Rudmik et al.^12^ conclude that the patient with a SNOT-22 score above 30 points have a 75% chance of significantly changing their clinical condition with surgery. These same patients improved their quality of life by 45%. On the other hand, patients with SNOT-22 scores under 20 did not show significant improvements after surgery.

In the present study, no statistically significant difference was found between the averages of the SNOT-22 score of the first consultation of patients for the groups that evolved to surgical or clinical referrals.

In a study that validated SNOT-22 to Portuguese, Kosugi et al.^8^ applied the questionnaire to 89 patients before and after sinonasal surgery and obtained an average preoperative score for the group with the disease of 62.39 compared to 49 + 19 of our sample.

In a prospective study, Mascarenhas et al.^13^ evaluated 60 patients with referrals for surgery prior to sinonasal surgery and obtained a score of 61.3 ± 24.

The present study was longitudinal and retrospective and the patients of this sample were initially treated clinically and referred for surgery during their ENT medical follow-up. Since collections were not carried out periodically or at the exact moment of the surgical referral, it is not possible to confirm whether the score deceased over time or whether the score of these patients was worse than the score of the first assessment at the time of the surgical referral.

The studies of Kosugi et al.^8^ and Mascarenhas et al.^13^ were conducted with patients with a confirmed surgical referral, which differs from the profile of our sample that did not have that confirmation.

In the Brazilian scenario, the difference found between the scores may also correspond to the fact that our sample used a service that attends private patients. This means that the studied subjects may have had a better socioeconomic status than the patients of the studies of Kosugi et al.^8^ and Mascarenhas et al.^13^ whose subjects used a public health service.

The expected pathophysiological rationale is that patients with referrals for surgery obtain higher scores and that this could explain the better scores of patients with referrals for clinical treatment. Soler et al.^8^ conducted a study with 242 patients analysed over time and found that patients selected for the surgical treatment obtained worse SNOT-22 scores than patients who chose clinical treatment. Factors such as demographic characteristics, patient-doctor relationship, comorbidities and personality did not influence the surgical outcome.

In the present study, there was no difference between the demographic characteristics of the groups. With respect to the doctor–patient relationship, the authors believe that the use of a single evaluator minimises this bias.

The outcome analysed in this study is the surgery indicated by the physician. This decision also depends on subjective factors, such as motivation, personal preference and expectations of the patients regarding the procedure. Of the patients of this study, four patients did not undergo surgery and decided to continue with clinical treatment.

The criterion for surgical indications was the failure of maximum clinical treatment after three weeks. Information from the actual patients regarding the absence of improvement in symptoms or even the worsening of symptoms and the will of the health professionals make selection more reliable and reduce the subjectivity of multiple observers.

The authors believe that these findings do not invalidate the information that the serial analysis and prospective follow-up of these patients can significantly enable a change of conduct and option for the right moment of surgical referral. Over time, considering the natural evolution of the disease or failure of clinical treatment with maintenance or worsening of scores of the questionnaire, this could lead to a significant difference between the groups that evolves to surgery to the detriment of clinical treatment.

Hopkins et al.,^14^ who validated the SNOT-22 for the first time in the United Kingdom, applied the questionnaire to 2077 surgical patients and obtained a preoperative score of 41.7, which is lower than the score found in the present study. This difference between the Brazilian studies and UK study suggests that the different lifestyles and cultures of the nations may influence the concept of quality of life. However, the UK sample of surgical patients consisted of subjects from several centres. Such a diverse criteria suggests that the sample included patients with few symptoms or a milder form of disease, which would be an error and may lead to over-referrals of surgical treatment.

Gillett et al.^15^ conducted a study and used the SNOT-22 on 116 patients without sinonasal disease in the United Kingdom to know the score of the questionnaire among patients without sinonasal disease. The justification was that many patients who underwent surgery in other studies obtained a relatively low SNOT-22 score, which suggests that the referral may have been inappropriate. Patients with low scores may have oligosymptomatic CRS or may have been overdiagnosed.

In our sample, patients were recruited from a single service and the referral was indicated by a single doctor, which minimises the risk of changes in criterion. Blinding in relation to the initial score also enables more robust data.

A limitation of this study is the non-discrimination of the CRS groups. We did not distinguish the subjects with sinonasal polyposis from the subjects with eosinophilia, for example. The intention was to help the otolaryngologist indicate surgical treatment irrespective of the type of disease. Furthermore, the size of the sample did not allow the creation of subgroups.

## Conclusion

Although this study did not include multiple and serial analysis, the first assessment showed that the SNOT-22 does not predict surgical outcome. It is therefore impossible to affirm whether these results over time, with serial assessments based on the questionnaire, could establish the SNOT-22 as a good decision-making tool.

## Conflicts of interest

The authors declare no conflicts of interest.
